# Topography influences megadune distribution and morphology

**DOI:** 10.1073/pnas.2531162123

**Published:** 2026-03-03

**Authors:** Hui Zhao, Keqi Wang, Yongwei Sheng, Deguo Zhang, Clément Narteau, Shengan Zhan, Fahu Chen

**Affiliations:** ^a^Key Laboratory of Ecological Safety and Sustainable Development in Arid Lands, Northwest Institute of Eco-Environment and Resources, Chinese Academy of Sciences, Lanzhou 730000, China; ^b^University of Chinese Academy of Sciences, Beijing 100049, China; ^c^Department of Geography, University of California, Los Angeles, CA 90095; ^d^School of Earth Sciences, Zhejiang University, Hangzhou 310058, China; ^e^Institut de physique du Globe de Paris, Université Paris Cité, CNRS, Paris 75238, France; ^f^Key Laboratory of Western China’s Environmental Systems (Ministry of Education), Lanzhou University, Lanzhou 730000, China; ^g^State Key Laboratory of Tibetan Plateau Earth System, Environment and Resources, Institute of Tibetan Plateau Research, Chinese Academy of Sciences, Beijing 100101, China

**Keywords:** megadunes, aspect ratio, topographic obstacle, shear stress, heterogeneity

## Abstract

Dunes are widely distributed on Earth and other extraterrestrial bodies, yet relatively little is known about what controls their maximum size. Earth’s megadunes (>100 m tall) have traditionally been attributed to constraints including atmospheric boundary layer depth, substrate bedrock type, and sediment supply. However, global mapping results presented here reveal that megadunes preferentially occur near mountains and within dunefield depressions. Megadune height-spacing transition from a power-law relationship to a near-normal distribution, and their aspect ratio (R_a_) with height shifts from inverse to direct proportionality. To investigate their underlying formation mechanisms, we focus on how topography influences megadune development under conditions of sufficient sand supply and constant wind regime, using a dune simulation model. Simulation results indicate that both positive (mountain-like) and negative (basin-shaped) topographies generate abrupt shear stress gradients, triggering rapid localized sand accumulation. Compared to gradual evolution on flat terrains, mountain-depression settings accelerate the dune coarsening process and megadune growth through enhanced sand flux convergence and increased collision rates between migrating dunes. Critically, surrounding topography modifies wind regimes, elevating dune aspect ratios (R_a_) as shear stress intensifies. Our proposed topography–aerodynamics–sediment redistribution mechanism for megadune formation on Earth and other extraterrestrial bodies demonstrates that terrain-induced wind regime heterogeneity is the fundamental control governing the formation and evolution of massive aeolian landforms.

## Significance

Global megadune distributions remain poorly characterized, with their formation mechanisms still debated. This study summarizes megadune distribution patterns at a global scale and finds that megadunes have distinct formation mechanisms and dynamic behaviors compared to normal-sized dunes. Simulation results reveal that both positive (mountain-like) and negative (basin-shaped) topographies induce abrupt shear stress gradients, which trigger rapid localized sand accumulation. In contrast to the gradual evolution observed on flat terrains, mountain–depression configurations accelerate dune coarsening and megadune development by enhancing sand flux convergence and increasing collision frequency among migrating dunes. This obstacle-driven evolution framework advances our understanding of aeolian sediment accumulation and megadune genesis across planetary surfaces.

Sand dunes abound in arid landscapes on Earth and other extraterrestrial bodies. While the mechanics of how sand dunes form on a flat bed of loose sediment are relatively well understood, much less is known about what controls their maximum size. Giant sand dunes, typically ranging from 20 to 450 m in height and spaced ~300 to 5,500 m apart, constitute the most striking landscapes in Earth’s deserts (1, 2). Similar impressive dune formations have also been identified on extraterrestrial bodies, such as Mars and Titan (3–6). The immense size, remote locations, and prolonged formation process of giant dunes create significant challenges to investigating their formation mechanisms through conventional field investigations (7–9). Aeolian sands can accumulate to form small-scale dunes within years, depending on wind patterns and regional geomorphology (10), while dunes exceeding 100 m in height, commonly known as megadunes (1, 11), often require over 10,000 y to reach their colossal dimensions (8). Based on relatively sparse datasets, a few studies indicate that the linear power-law relationship between dune height and spacing weakens as dune size increases (12–14). A recent global data using an average of 32 × 32 km^2^ grids in desert regions further reveals that this relationship departs from a power-law pattern in the highest dune area, which exceeds ~100 m (15). This scaling transition indicates a natural distinction between small dunes and megadunes. Given the coarse resolution of existing datasets (12–15), we adopt 100 m as a conservative threshold for defining terrestrial megadunes, noting that this threshold may vary for extraterrestrial bodies.

A simple dunefield construction can be considered as the accumulation and amalgamation of individual normal-sized typical dunes (16–19), formed through self-organization processes forced by wind regime, sediment supply, source-area geomorphology and vegetation conditions (20–26). Though the formation of megadunes follows the same physical principles as small-scale dunes, the critical question remains: “what factors and mechanisms control the size of megadunes?” Investigating the formation mechanisms of megadunes can provide valuable insights into sediment accumulation and the self-organization processes that shape dunefields.

A previous study suggested that dune size is determined by the average depth of the atmospheric boundary layer (ABL), according to the regional topography and atmospheric conditions (27). However, a recent study on dune size in 32 × 32 km^2^ grids covering global dunefields, using remote sensing images and numerical experiments, concluded that the height of megadunes may increase indefinitely but is limited mainly by sand supply (15). The mechanisms controlling the formation and size of megadunes remain controversial.

Although megadunes are widely distributed on Earth, their morphologies are complex, and their distribution and underlying geographical environments are highly variable (2, 22, 23). Moreover, our knowledge of the distribution and morphology of individual megadunes at global scale is limited. Examining the distribution, morphology, and environmental context of megadunes enables us to summarize their patterns and regularities and to investigate their formation mechanisms. Dunefields are located mainly in nine regions worldwide (28) (*SI Appendix,* Fig. S1). In this study, we map individual megadunes in these regions and classify them into four main types according to their morphologies using geospatial information technologies (*Materials and Methods,* *SI Appendix,* Figs. S2 and S3). These four dune types are dome-shaped dunes (D_d_), without a dominant slip face; transverse dunes (D_t_), with one main slip face; longitudinal dunes (D_l_), with bidirectional main slip faces; and star dunes (D_s_), with multidirectional slip faces (*SI Appendix,* Table S1). We then establish a global megadune database (Dataset S1) containing critical parameters including location, height, and spacing for nearly 56,000 megadunes; summarize the distribution patterns of global megadunes; analyze the possible factors affecting the distribution and development of megadunes; and finally verify the mechanisms of the formation and distribution of megadunes using model simulations.

## Results and Discussion

### Global Megadune Distribution.

**Fig. 1.**
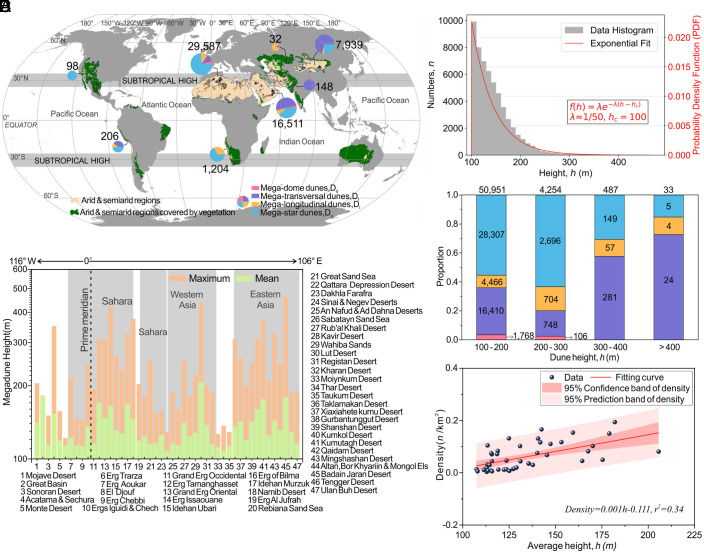
Distribution of global megadunes revealed by the database. (*A*) Distribution of global megadunes. Global megadunes are organized into eight subregions based on geographic location with the categorized quantities in each subregion displayed in the pie chart. Megadunes with different morphologies (D_d_, D_t_, D_l_,D_s_) are colored pink, lavender, orange, and blue, respectively. (*B*) Maximum and mean height of megadunes within each desert, arranged from west to east. (*C*) Frequency histograms of megadunes in various height classes and PDF fit. (*D*) Distribution proportion of megadunes for the four dune types (D_d_, D_t_, D_l_, D_s_) in four major height classes. (*E*) Scatterplot of mean megadune height *h* versus megadune density (*n*/km^2^) for each megadune field.

The established global megadune database contains 55,725 megadunes taller than 100 m across the world’s deserts/dunefields (Fig. 1 *A* and *B*). The majority (>97%) of these megadunes are concentrated in the Sahara (29,587) and Arid Asia (24,630), influenced by subtropical high pressure, while the remainder (<3%) are scattered across other regions (Fig. 1*A*). The arid climate and associated lack of vegetation cover in these regions are essential for the genesis and expansion of the vast desert area that provides sufficient aeolian sand supply for megadune formation (28). Despite the vast expanse of deserts in Australia, megadunes are notably absent because of the relatively high vegetation cover and low sand drift (29, 30). From Fig. 1*B*, which shows the distribution of megadune deserts from west to east, it is evident that megadunes tend to be taller in the hinterland of the Sahara and Arid Asia.

We found that the megadune quantity probability decreases exponentially versus height *h* with a probability density function (PDF): f (*h*) ≈ 1/50 exp (−(*h* − *h*_c_)/50), where *h_c_* = 100 m (Fig. 1*C*). Specifically, each 100 m increase in dune height results in a one-order of magnitude reduction in dune number. Most of the inventoried megadunes (50,951) are between 100 m and 200 m tall and are dominated by the D_s_ morphology category (3% for D_d_, 32% for D_t_, 9% for D_l_, and 56% for D_s_), suggesting that multidirectional sand-drift winds are the most conducive to the development of these megadunes (15) (Fig. 1*D*). A total of 4,254 megadunes reach heights between 200 m and 300 m, while 487 megadunes have heights of 300 to 400 m. Only 33 megadunes over 400 m in height are found in three distinct dunefields: the Badain Jaran Desert in China, the Lut Desert in Western Asia, and the Erg Issaouane in the Sahara. Megadunes over 300 m tall are dominated by the D_t_ category, formed under a unidirectional wind regime, and no D_d_ megadunes can attain a height over 300 m. Fig. 1*E* plots the population density of megadunes (defined as the number of megadunes per km^2^ in each megadune field) (*SI Appendix,* Table S2). This reveals an increasing trend with increasing mean height, suggesting that taller megadunes tend to cluster together.

**Fig. 2.**
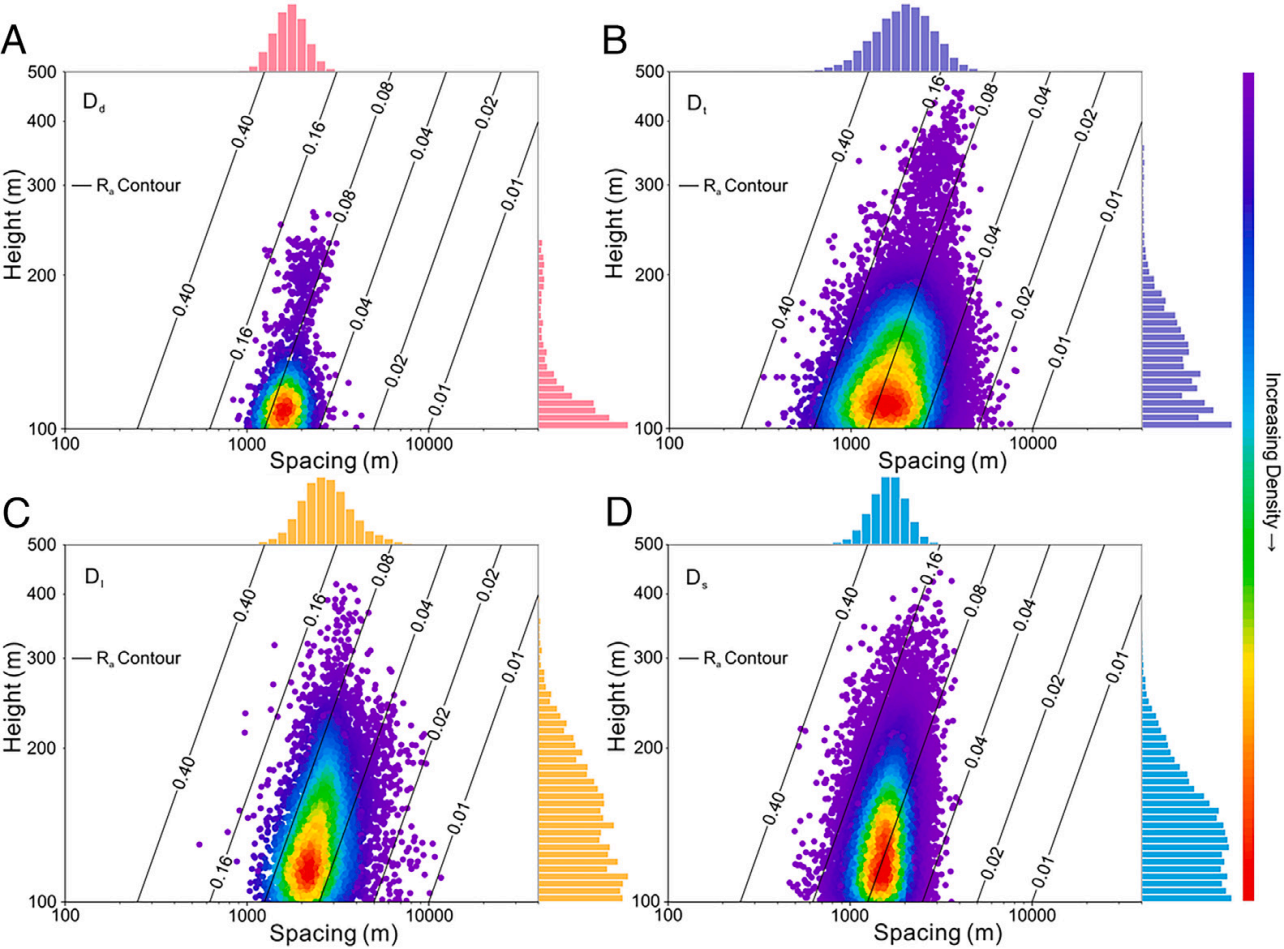
Height and spacing of global megadunes. (*A*–*D*) Relationship between height and spacing for D_d_, D_t_, D_l_, and D_s_, respectively. Points are colored based on density using the color bar on the right. Histograms of dune spacing and height are along the top and right side of each plot. R_a_ contour lines suggest that taller megadunes tend to have higher R_a_.

We found that no power-law relationship is evident between megadune height and spacing, as described in previous local-scale studies (12–14). A scatterplot of megadune height and spacing shows a high dispersion between megadune spacing and height (Fig. 2), negating the previously claimed power-law relationship. As the height of megadunes increases, the distribution of dune spacing decreases, converging around a maximum likelihood spacing (Fig. 2). The number of megadunes in each morphological type decreases exponentially with height. The overall distribution of dune spacing for each geomorphic type approximates a normal distribution.

The aspect ratio (R_a_), defined as the ratio of dune height to spacing (*h/s*), is a key characteristic of dune morphology. To elucidate the relationship between R_a_ and dune height, we added R_a_ contour lines (Fig. 2) and found that larger megadunes exhibit higher R_a_ values. Contrary to previous studies suggesting a power-law relationship (12–14), the relationship between dune height and spacing deviates significantly from a power law when dune height exceeds 60 m (*SI Appendix*, Fig. S4*A*), based on one height-spacing dataset of an average of 32 × 32 km^2^ within global sand seas (15). We extracted height-spacing data for over 100,000 dunes from major deserts worldwide. Based on these data, we found that the deviation from a power law occurs at a dune height of ~80 m (*SI Appendix*, Fig. S4*B*). R_a_ contour analysis further reveals a dichotomy in dune behavior: Smaller dunes exhibit an inverse relationship between R_a_ and height, while taller dunes exceeding ~60/80 m demonstrate a positive correlation (*SI Appendix*, Fig. S4). These distinct patterns suggest fundamentally different formation mechanisms and dynamic behaviors between megadunes and normal-sized dunes. Considering the overlap of data from dunes with varying morphology characteristics, these results further validate the rationality of selecting 100 m as the height threshold for classifying megadunes on Earth. Although remote sensing images show dunes exceeding 100 m in height on Mars and Titan (3–6), determining whether they should be classified as megadunes would still require the compilation of dune height-spacing data across extraterrestrial bodies to establish an appropriate height threshold for megadunes on these bodies.

### Proximity to Mountains.

Observing that the megadune-occupied hinterlands of the Sahara and Arid Asia commonly feature major mountain ranges (Fig. 1*B* and *SI Appendix,* Fig. S5), we further analyzed our database in the context of mountain distributions. This indicates that megadunes preferentially form in areas adjacent to mountain ranges. To explore the relationship between megadune distribution and proximity to mountains, we measured the distance of each megadune from its nearest mountain ridge (*Materials and Methods*). The correlation between megadune height and distance to mountains reveals that megadune height initially increases as distance decreases, but then decreases beyond a certain distance (*SI Appendix,* Fig. S6). Combining data from all dunefields, we observe a clear trend: Megadune height increases significantly near mountains, with two concentrated distribution zones around <100 km (~48%) and 150 to 250 km (~29%) from mountains (*SI Appendix*, Fig. S7*A*). In contrast, megadunes are rare beyond distances of 350 km, and all >250 m megadunes are located within 100 km of mountains. Further analysis reveals that dune spacing decreases with increasing proximity of megadunes to mountains (*SI Appendix,* Fig. S7*B*). Furthermore, regardless of dune type (D_d_, D_t_, D_l_, D_s_), dunes close to mountains tend to be taller, have a smaller spacing, and are concentrated primarily within two zones: <100 km and 150 to 250 km (*SI Appendix,* Fig. S7).

**Fig. 3.**
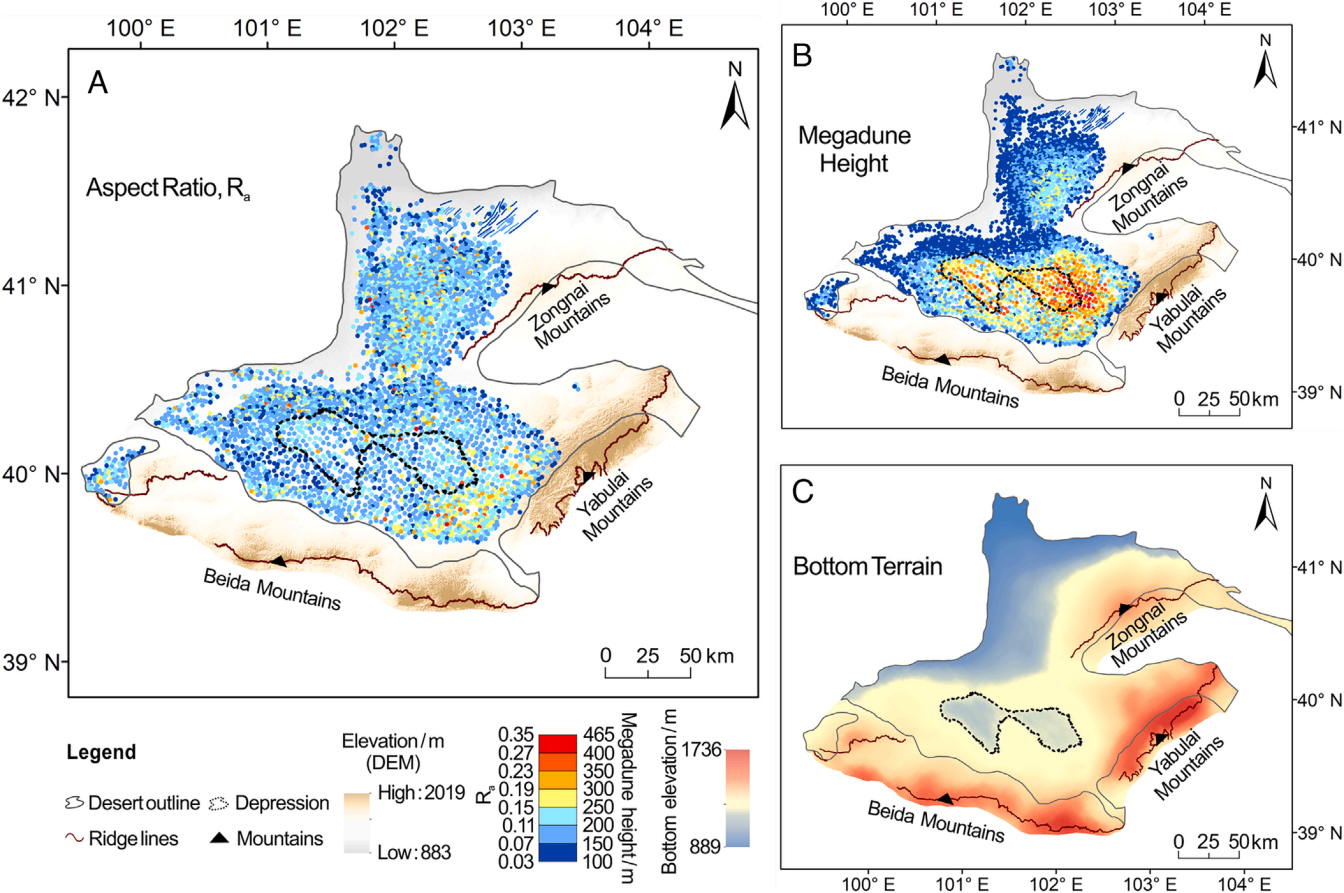
Sampled dunefield (the Badain Jaran Desert), demonstrating the influence of depression/mountain landforms on megadune development. (*A* and *B*) Distribution of aspect ratio (R_a_) and megadune height in the Badain Jaran Desert, respectively. (*C*) Topographic features of the dune bottoms in the Badain Jaran Desert. Data are derived from spatial interpolation performed on the bottom points within the dunefield.

As demonstrated in the Badain Jaran Desert (Fig. 3*A*), megadunes in proximity to mountainous regions consistently exhibit elevated R_a_ values, regardless of their morphological classification—a pattern that is substantiated by observations from other megadune fields (*SI Appendix,* Fig. S8). Notably, our analysis reveals a spatial disjunction between areas containing taller megadunes and those displaying higher R_a_ values (Fig. 3 *A* and *B* and *SI Appendix,* Fig. S8). Specifically, regions with high R_a_ values are predominantly located close to mountainous areas, suggesting a potential topographic influence on dune morphology. An aeolian geomorphology study of Mars also concluded that topography influences aeolian fluxes and dune morphology development (31).

### Megadunes in Depressions.

We also examined the basal terrain of each dunefield by connecting the base elevation of each megadune. Most dunefields have a relatively flat terrain, except for three deserts (the Idehan Ubari, the Rub’ al Khali, and the Badain Jaran deserts), with depressed topography ~30 to 70 m deep (Fig. 3*C* and *SI Appendix,* Fig. S8 *C* and *F*). A large proportion of tall megadunes in these dunefields are located in depressions (*SI Appendix,* Table S3). The depressions in these three dunefields cover <10% of the total area of global megadune fields, yet they host nearly 20% of the megadunes. Specifically, ~31% of megadunes exceeding 300 m in height and ~33% of those exceeding 400 m occur within these depressions. This suggests that a depression terrain also favors megadune development.

This is especially so for the Rub’ al Khali Desert (*SI Appendix*, Fig. S8 *D*–*F* and Table S3), where a 78 m deep depression covers ~87,300 km^2^ (~50% of the area of the megadune field), where over 61% and 97% of the megadunes over 100 m and 200 m tall, respectively, are developed. Most areas of the depression are ~300 km away from the surrounding mountains, and thus it appears that the depressions also have their own mechanism for megadune development.

### Mountain and Depression Forcing of Megadune Patterns.

**Fig. 4.**
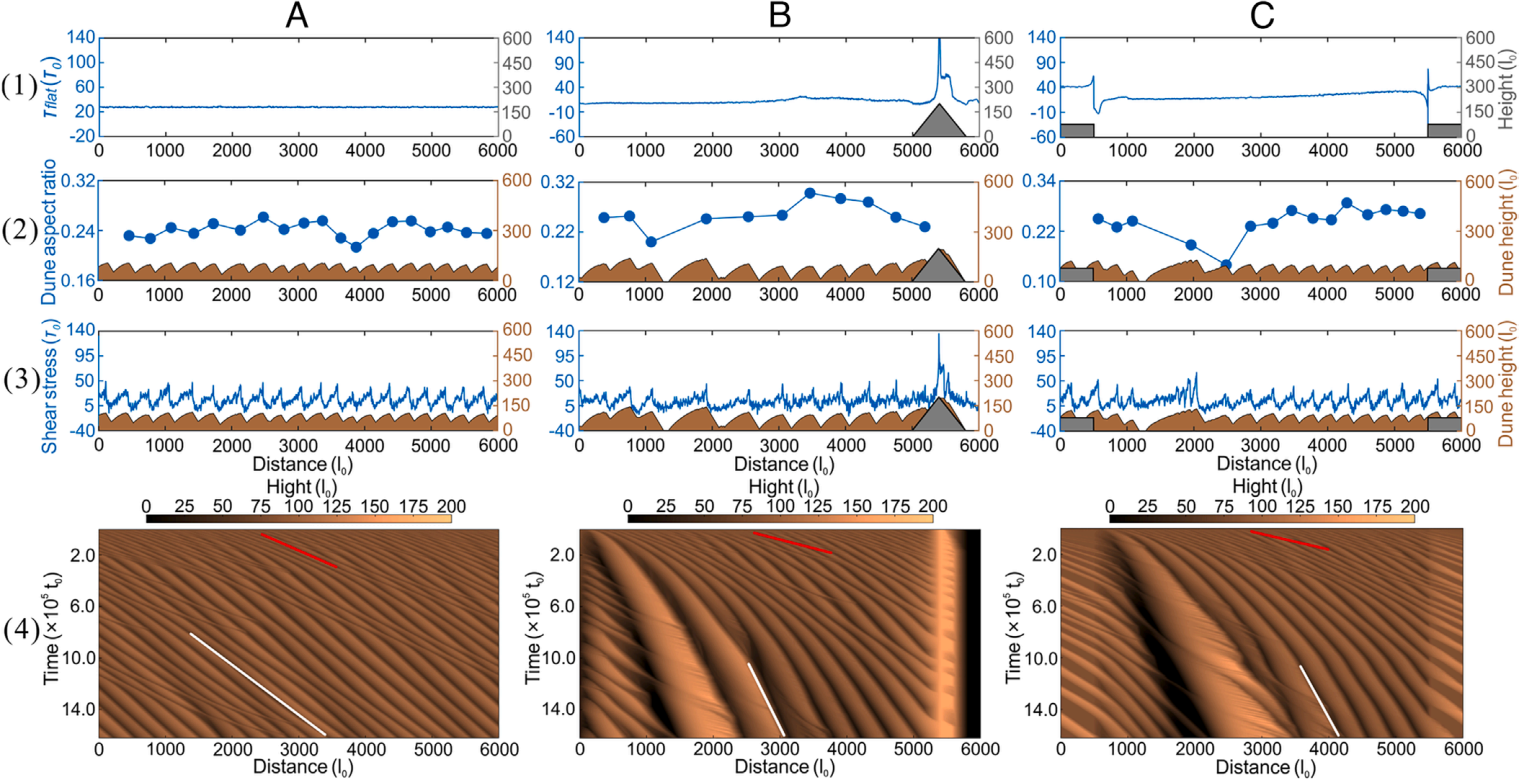
Numerical simulation of the evolution of dunefield patterns under three typical boundary conditions, with the same starting conditions of sand layer, wind regime, and flow depth. (*A*) Flat, obstacle-free bed, (*B*) Mountain-like positive topography, and (*C*) Basin-like negative topography. (1) Initial shear stress distribution for each of the three scenarios without a sand layer. (2) Aspect ratio (R_a_) of each dune for each of the three scenarios. (3) Average shear stress for each scenario when dunes have formed. (4) Temporal evolution of dunefields. Slopes of the red and white lines indicate the migration rate of the dunes at early and later stages in each scenario. Dunes in a flat-bed have the same migration rate, while in positive and negative, scenarios the dunes have heterogeneous migration rates in early stages.

Model simulation confirms that the spatial organization of megadune fields is closely linked to large-scale topographic obstacles such as mountains and depressions. To isolate the role of these features, we performed three sets of numerical experiments with identical initial conditions—a sand layer 80 l_0_ thick, a unidirectional wind regime, and the same flow depth. We only varied the underlying topography: i) a flat, obstacle-free bed, ii) mountain-like positive relief, and iii) basin-like negative relief (Fig. 4). All runs were continued until the dunes attained a mature, quasisteady configuration.

On the flat bed, dunes rapidly approached a steady state characterized by a spatially uniform surface shear stress that governed the uniform dune shape and height (Fig. 4*A*). In contrast, the presence of a mountain-like positive topography significantly disrupted this uniformity and accentuated the shear-stress heterogeneity, and the dunes also migrated heterogeneously (Fig. 4 *B*4), compared with the flat-bed with rapid coarsening. This coarsening process significantly reduces the number of dunes in the dunefield. With the same total sand volume distributed among fewer dunes, taller dunes formed compared to those on a flat sandy bed. Previous work demonstrated that *R*_a_ = *h/s* varies with flow strength (32). As airflow approached the obstacle, shear stress generally increased and this acceleration leads to higher R_a_ values and taller dunes near obstacles (Figs. 3 and 4*B*). Due to the heterogeneity of the dunefield, the coarsening rate of dunes in certain locations significantly surpasses that in other areas and dunes in these localized zones become substantially larger and capture more sand. Consequently, the largest megadunes do not form at the highest R_a_ locations close to mountains, due to limited sediment availability (Figs. 3 and 4*B*).

In the basin-like negative topography scenario, shear stress heterogeneities also exert a significant influence on aeolian sediment transport processes and thereby govern the initiation, development, and interaction dynamics of bedforms. Simulations at the initiation stage demonstrate that a localized reduction in shear stress, which occurs as the bed transitions from a consolidated to an erodible bed, amplifies sand deposition and triggers the development of dunes at specific sites (Fig. 4*C*), while subsequent stress recovery facilitates rapid dune growth. Simultaneously, the heterogeneity of the dunefield modifies the spatial distribution of surface shear stress across the dunefield and accelerates interactions between dunes, including collision, merging, and dune coarsening dynamics (Fig. 4 *C*4). In the area close to the downwind edge of the depression terrain, topographic elevations simultaneously impede dune migration and accelerate the local wind regime, promoting sand accumulation and progressively increasing both dune height and the associated surface shear stress, ultimately yielding larger *R_a_* values.

In the above three simulation scenarios, we focused exclusively on the influence of topography on dune development, whereas the respective roles of wind forcing and sand supply remain unclear. We conducted a suite of numerical simulations to systematically explore dune-field evolution under different wind strength and sand availability (*SI Appendix,* Fig. S9) in both flat sand fields and dunefields with obstacles. The simulation results demonstrate that both abundant sand supply and strong sediment-transporting winds promote dune height increase. However, wind forcing exerts a more pronounced control on dune height development than sand availability. Comparisons between flat and obstacle scenarios further reveal that, regardless of the combination of wind strength and sand supply, dunes forming in the presence of obstacles consistently attain greater mean heights than those developing on flat sand surfaces (*SI Appendix,* Fig. S9).

Collectively, these simulations demonstrate that both positive and negative topographic features systematically promote dune coarsening by inducing persistent spatial heterogeneity in wind regime and sediment distribution. Where sand supply and wind strength are sufficient, such heterogeneity destabilizes the uniformity of dune migration, accelerates the dune coarsening process, and concentrates sediment into progressively fewer but larger dunes. In contrast, flat dunefields experience nearly uniform shear stress and sediment flux, leading to synchronous dune migration and dune development. Under such homogeneous conditions, dune coarsening is suppressed because sand is evenly distributed among dunes.

This study represents an initial simulation of only the two most fundamental and universal types of positive and negative topographies and their influence on megadune formation. Our analysis does not address more complex terrain configurations, such as dunefields between two mountains or in V-shaped deep basins (33). These scenarios are all combinations or variations on the fundamental scenarios we have studied and they require substantial future investigation. Nevertheless, our work establishes a conceptual framework for understanding loose sediment accumulation processes.

## Conclusions

This study is a global-scale investigation of megadune distribution. It establishes a definition of megadunes, summarizes their distribution regularities, and identifies the key factors that govern dune size development, using model simulations. The surrounding mountains and depressions in some dunefields play a crucial role in the self-organization of dunefields, facilitating the rapid coarsening of dunes and changing their morphological features. Notably, megadunes are not exclusive to Earth but may also exist on other extraterrestrial bodies in the Solar System, such as Mars and Titan (3–6). Our findings offer a perspective on the processes driving the accumulation of loose sediments on Earth and other planets.

## Materials and Methods

### Dune Height and Spacing Extracted by Triangulated Irregular Network (DHSET).

Megadunes are mapped from global digital elevation models (ASTER GDEM V2) (34) using geospatial information technologies. A tool, called the DHSET, was developed to locate sand dunes in a dunefield and extract their height and spacing. All the top and bottom points of dunes within a dunefield are extracted using focal statistics tools in ArcGIS software (35). The top points of dunes are used to establish the TIN (36) model. Multiple triangles are formed around each dune top, with bottom points distributed within these triangles (*SI Appendix,* Fig. S3 *B*, *E*, *H*, and *K*). Megadunes are divided into four morphological types (D_d_, D_t_, D_l_, D_s_) based on the commonly used morphological principle (2, 37–45) (*SI Appendix*, Fig. S2 and Table S1). Dune height is extracted by calculating the elevation difference between its top point and the bottom point within these triangles. Dune spacing is calculated as the geometric distance from the dune top point to the opposite side of the triangle in the downwind direction (yellow arrows in *SI Appendix,* Fig. S3 *B*, *E*, *H*, and *K*).

For each transverse dune (D_t_) top point, there is only one height value and one spacing value (*SI Appendix,* Figs. S3 *D*–*F*). For each longitudinal dune (D_l_) top point, there are two height values and two spacing values, respectively, for their double downwind direction (*SI Appendix,* Figs. S3 *G*–*I*). For each dome (D_d_) or star (D_s_) dune top point, there are multiple height values and spacing values, involving multiple downwind directions (*SI Appendix,* Figs. S3 *A*–*C* and *J*–*L*). The extracted height and spacing for each D_l_, D_s_, or D_d_ top point is the average of the height and spacing values involved. For D_l_ with several top points on a single dune, the maximum height and spacing are selected as the dune’s height and spacing (*SI Appendix,* Fig. S3*H*). The established global megadune database (GMDD) contains the location, height, and spacing properties of nearly 55,725 individual megadunes worldwide.

### Distance from Megadunes to Mountains.

The distance from megadunes to mountains was calculated based on ASTER GDEM V2 data (34) using ArcGIS (35). First, we identified the ridge lines of mountains using the Hydrology Analysis module, and then used the Near-tool to calculate the distance from the top point of megadunes to the closest mountain ridge line.

### ReSCAL Dune Simulation Model.

The real-space cellular automaton (ReSCAL) dune model (16) has been widely used in investigating the genesis and migration of various dune types, including barchan, transverse, star, and reversing dunes. This model not only provides valuable insights into the morphodynamical features of these dunes but also offers a unique perspective on the migration history of sediment particles (46) and changes in stratification patterns (47, 48). By integrating sediment transport simulations with airflow dynamics, the ReSCAL model leverages cellular automaton principles to capture the intricate interplay between sediment and air. Nearest-neighbor interactions govern sediment processes such as erosion, deposition, and transport, while lattice-gas cellular automaton simulations capture the complex feedback between wind stress and dune morphology. For a comprehensive overview, see Narteau et al. (16).

## Supplementary Material

Appendix 01 (PDF)

Dataset S01 (XLSX)

## Data Availability

The data generated in this study, such as megadune height, spacing, are provided in the form of an Excel database located in the supporting information. The ASTER GDEM V2 data (34) used in this study is provided by the Geospatial Data Cloud site, Computer Network Information Center, Chinese Academy of Sciences (http://www.gscloud.cn). Other data are included in the article and/or supporting information.
