# Supplementary material for: Topography influences megadune distribution and morphology
Source: Proc Natl Acad Sci U S A. 2026 Mar 3;123(10):e2531162123. doi: 10.1073/pnas.2531162123 (PMC12974449; doi:10.1073/pnas.2531162123)
Supplement: Supplementary file 1 — Appendix 01 (PDF) [file pnas.2531162123.sapp.pdf]

PNAS

Supporting Information for

Topography influences megadune distribution and morphology

Hui Zhao[a,1], Keqi Wang[a,b,1], Yongwei Sheng[c,2], Deguo Zhang[d,2], Clément Narteau[e], Shengan Zhan[c], Fahu Chen[f,g,2]

Corresponding author: Yongwei Sheng, Deguo Zhang or Fahu Chen
Email: ysheng@geog.ucla.edu, dgzhang@zju.edu.cn, or fhchen@itpcas.ac.cn.

This PDF file includes:

Figures S1 to S9
Tables S1 to S3
Legends for Dataset S1
SI References

Other supporting materials for this manuscript include the following:

Dataset S1

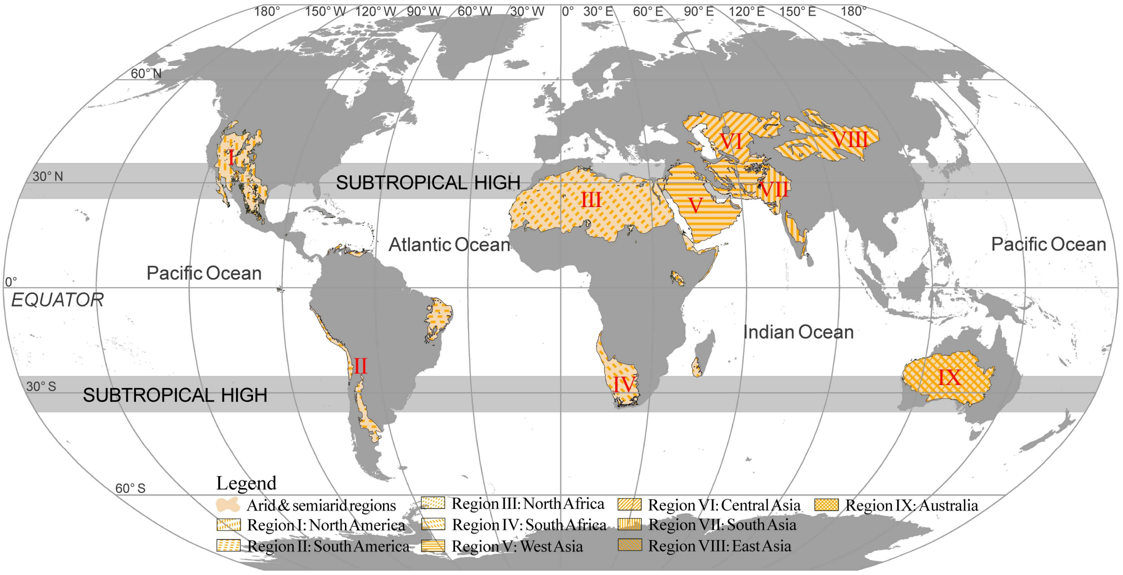

Fig. S1. Map of global arid and semiarid regions (modified from ref.1). Global arid and semiarid regions are divided into nine subregions based on their location.

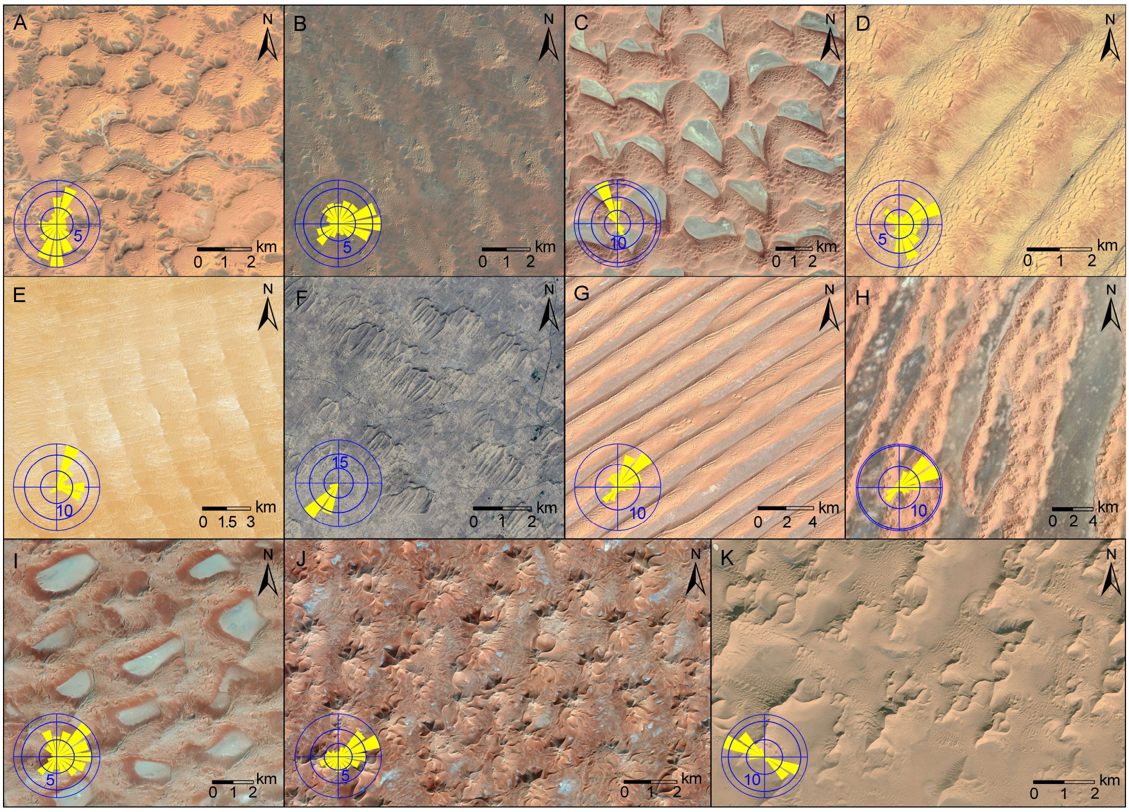

Fig. S2. Morphological characteristics of typical megadunes from satellite images (*Google Earth*). (A) Compound dome megadunes, Ad Dahna Desert (44°27′E, 25°50′N). (B) Complex dome megadunes, Grand Erg Occidental (2°35′E, 31°57′N). (C) Mega-barchans, Rub'al Khali Desert (54°34′E, 22°22′N). (D) Compound transverse megadunes, Idehan Ubari (13°28′E, 26°58′N). (E) Complex transverse megadunes, Erg of Bilma (12°3′E, 17°35′N). (F) Parabolic megadunes, Thar Desert (72°14′E, 26°21′N). (G) Compound longitudinal megadunes, Rub' al Khali Desert (48°53′E, 18°21′N). (H) Complex longitudinal megadune, Grand Erg Oriental (4°55′E, 29°16′N). (I) Network megadunes, Idehan Murzuq (13°01′E, 25°08′N). (J) Stellate megadunes, Grand Erg Oriental (8°44′E, 30°13′N). (K) Pyramid megadunes, Badain Jaran Desert (102°32′E, 39°41′N).

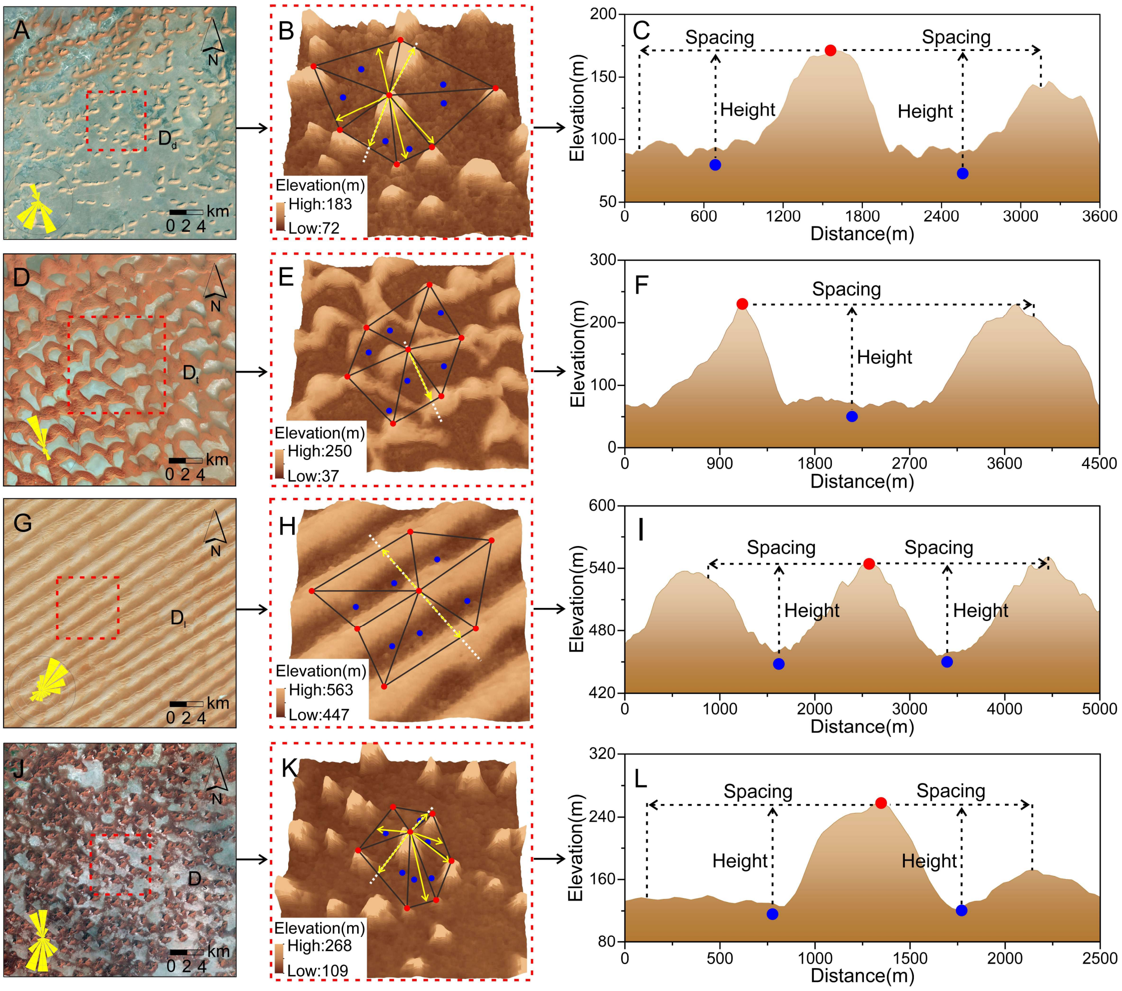

Fig. S3. Extraction of dune height and spacing. Panels (A-C), (D-F), (G-I) and (J-L) are examples of DHSET for four dune types (D_d, D_t, D_l, D_s) respectively. (A, D, G, J) Landsat images with a window size of 0.25°×0.25° showing the morphologies of D_d, D_t, D_l and D_s dunefields. (B, E, H, K) Sketch map of the DHSET. For the target and the surrounding adjacent dunes, the lines connecting the top points (red dots) of each dune form a triangular irregular network (TIN). The bottom points (blue dots) are distributed within the triangles of the network. Height and spacing calculations are given in Materials and Methods. (C, F, I, L) Sketch maps of profiles (white dashed lines) in panels (B, E, H, K) showing the height and spacing calculation for target megadunes.

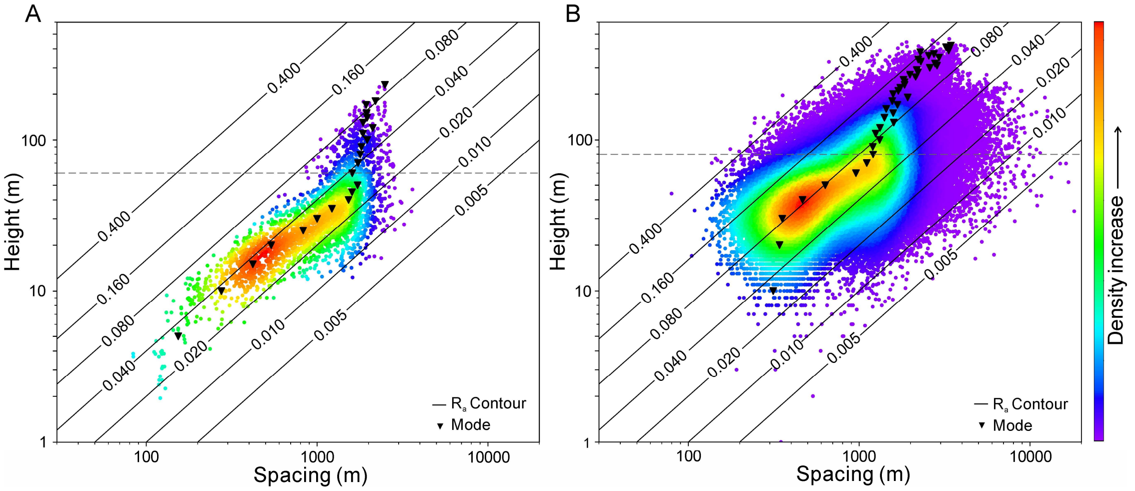

Fig. S4. Trend of dune height versus dune spacing. (A) Global dune morphology of an average 32×32 km^2 within global sand seas (redrawn from ref. 2). (B) Morphologies of over 100,000 dunes from the eight largest deserts on Earth. Point colors indicate relative data density in two-dimensional parameter space derived from Gaussian kernel density estimation. Black triangles mark the mode of the dune spacing distribution at different heights, and black parallel lines represent the contours lines of R_a. The height-spacing relationship deviates from a power law when dune heights exceed 60 m for (A) and 80 m for (B). R_a contour lines suggest that for smaller dunes, R_a decreases with increasing height, while R_a increases with increasing height when dune height exceeds 60 m for (A) and 80 m for (B).

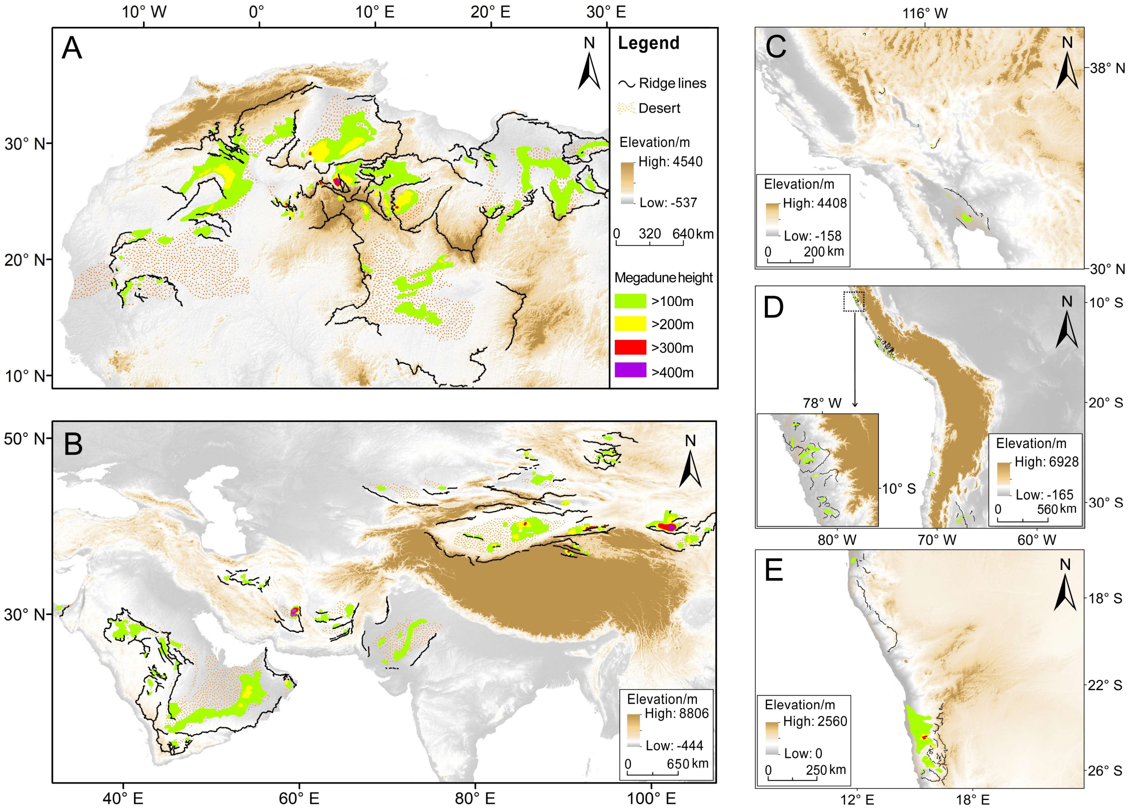

Fig. S5. Distribution of megadune fields adjacent to mountains. (A-E) Distribution of megadunes with different heights in North Africa (A), Asia (B), North America (C), South America (D), South Africa (E).

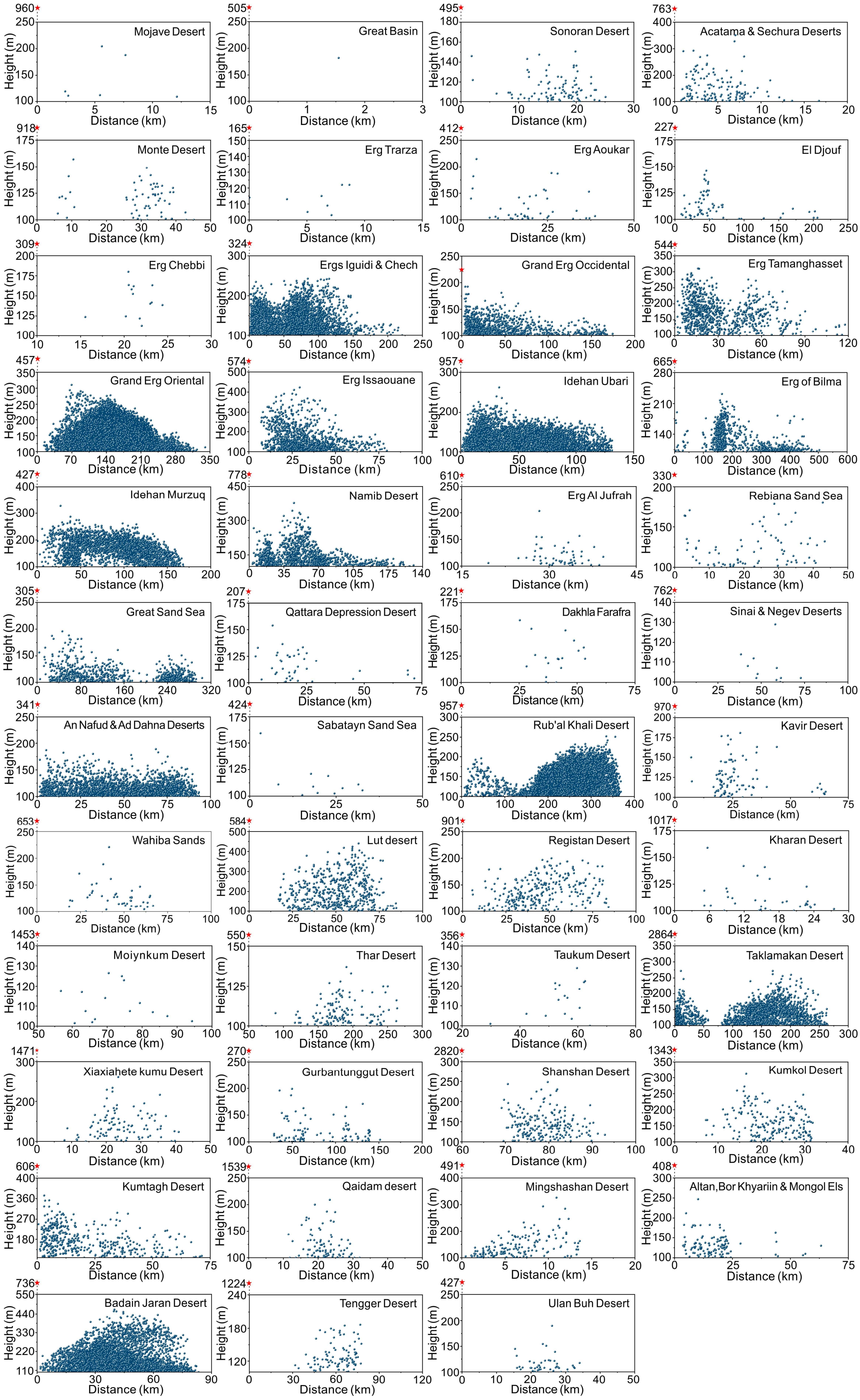
Mojave Desert
Great Basin
Sonoran Desert
Acatama & Sechura Deserts
Monte Desert
Erg Trarza
Erg Aoukar
El Djouf
Erg Chebbi
Ergs Iguidi & Chech
Grand Erg Occidental
Erg Tamanghasset
Grand Erg Oriental
Erg Issaouane
Idehan Ubari
Erg of Bilma
Idehan Murzuq
Namib Desert
Erg Al Jufrah
Rebiana Sand Sea
Great Sand Sea
Qattara Depression Desert
Dakhla Farafra
Sinai & Negev Deserts
An Nafud & Ad Dahna Deserts
Sabatayn Sand Sea
Rub'al Khali Desert
Kavir Desert
Wahiba Sands
Lut desert
Registan Desert
Kharan Desert
Moiynkum Desert
Thar Desert
Taukum Desert
Taklamakan Desert
Xiaxiahete kumu Desert
Gurbantunggut Desert
Shanshan Desert
Kumkol Desert
Kumtagh Desert
Qaidam desert
Mingshashan Desert
Altan,Bor Khyariin & Mongol Els
Badain Jaran Desert
Tengger Desert
Ulan Buh Desert
Height (m)
Distance (km)

Fig. S6. Relationship between megadune height and distance to mountains. In each desert, there exists one optimal distance where megadunes have a maximum height. The red dot on each Y axis represents the relative elevation difference between the base of the tallest megadune and its corresponding mountain ridge. This indicates that the highest megadune in each megadune field is below the surrounding mountain ridge.

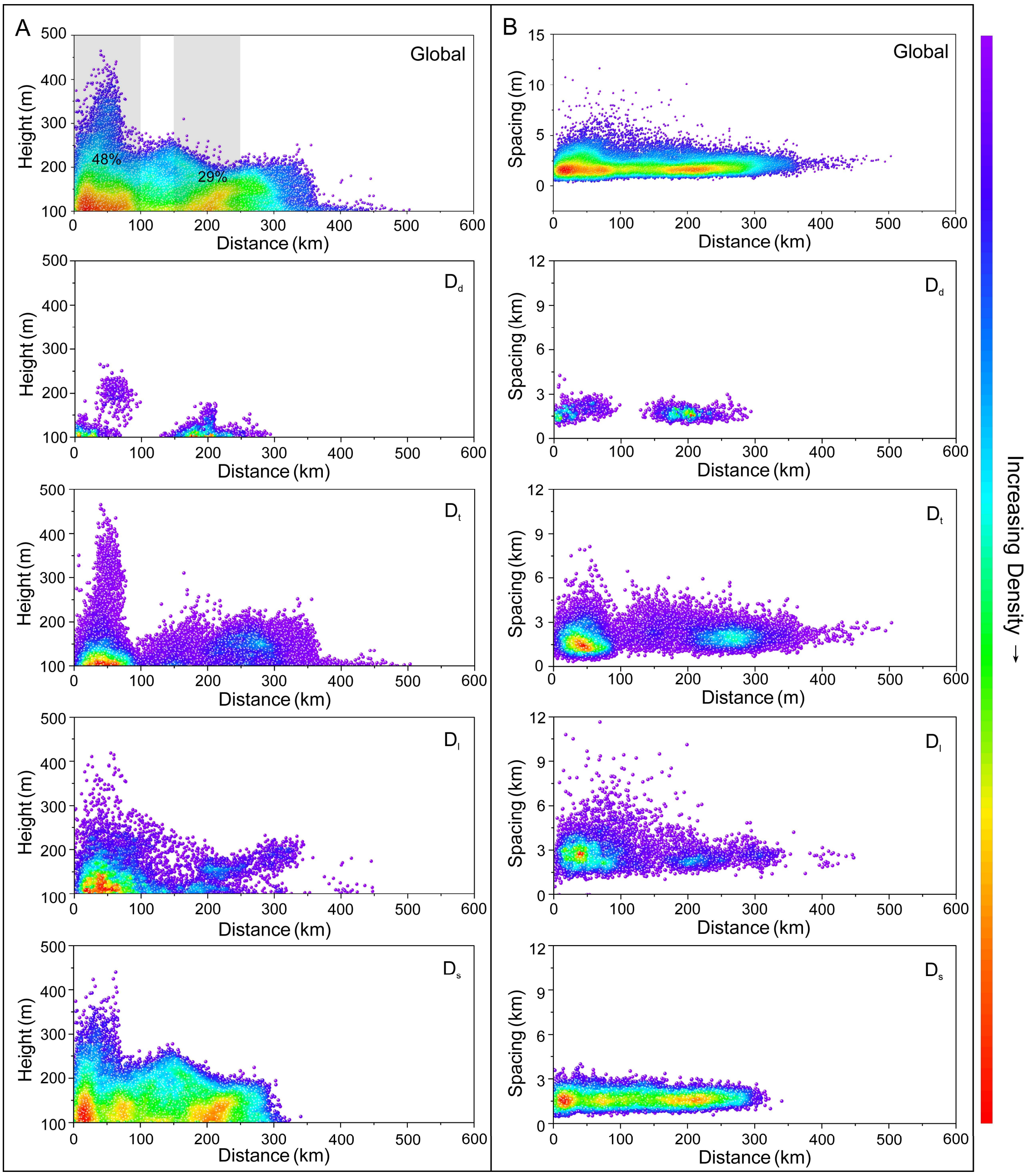

Fig. S7. Relationship between morphology and distance to mountains for global megadunes. (A) Kernel density plot between dune height and distance to mountains for global, D_d, D_t, D_l, and D_s megadunes, respectively. (B) Kernel density plot between dune spacing and distance to mountains for global, D_d, D_t, D_l and D_s megadunes, respectively.

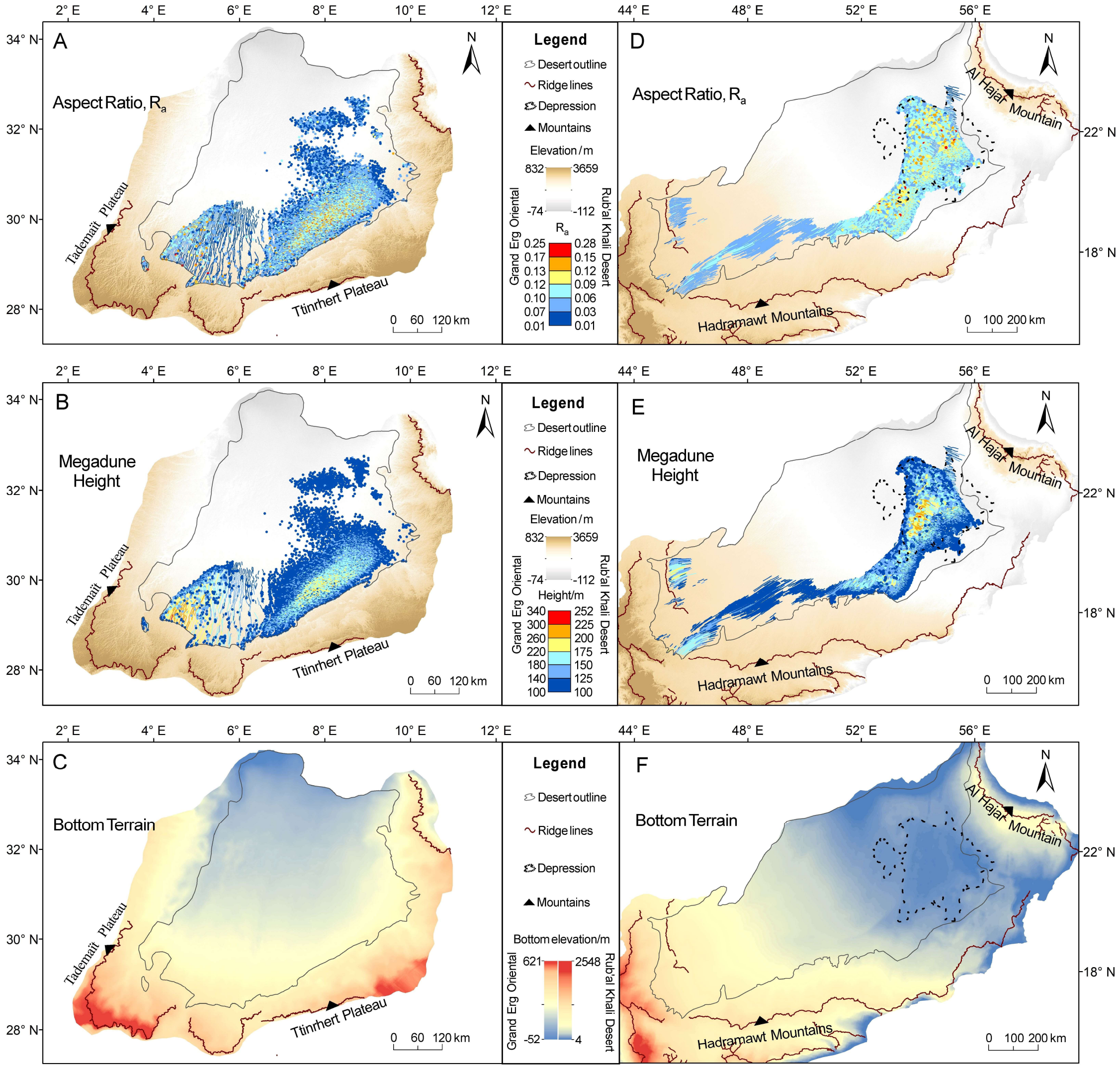

Fig. S8. Examples of global megadune fields under two scenarios: with and without depressions. (A-C) Distribution of aspect ratio (R_a), height and bottom topography in the Grand Erg Oriental. (D-F) Distribution of aspect ratio (R_a), height and bottom topography in the Rub' al Khali Desert. There is an obvious depression terrain in the Rub' al Khali Desert.

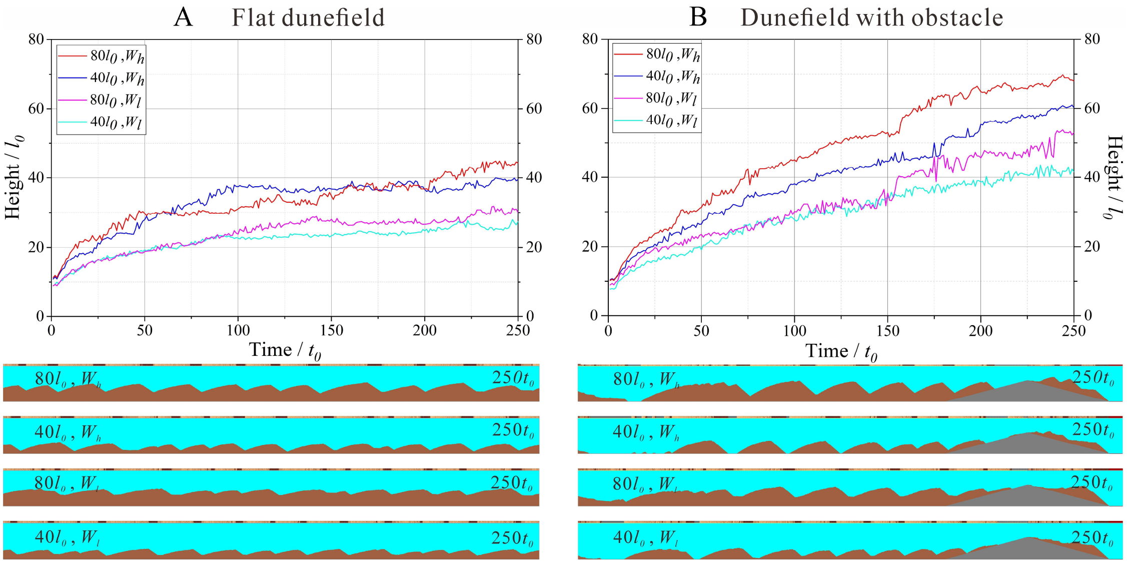

Fig. S9. Numerical simulation of flat dunefields (A) and dunefields with an obstacle (B). Different sand layer, thicknesses and wind speeds are used: high wind speed (W_h) with a thick sand layer ($80l_0$); high wind speed (W_h) with a thin sand layer ($40l_0$); low wind speed (W_l) with a thick sand layer($80l_0$); and low wind speed (W_l) with a thin sand layer ($40l_0$).

Table S1. Tentative classification system of global megadunes. The classification system is based mainly on morphology. Megadunes are divided into four categories based on the number of slipfaces, and then further classified into 11 secondary types. The classification of each megadune type is a composite representation derived from megadune images (Fig. S2) and definitions from the literature.

Number of slipfaces	Morphological type		Morphological description	Corresponding figure	Ref.
	Primary	Secondary			
0	Dome megadune	Compound dome megadune	Circular or elliptical shaped dunes with no obvious slip face and a flat mesa. Deformation forms include triangular domes, insects-like domes, etc.	Fig.S2 A	3-6
		Complex dome megadune	Circular or elliptical shaped dunes, with other types of sand dunes stacked on a relatively flat mesa.	Fig.S2 B	3-6
1	Transverse megadune	Mega-barchan	Crescentic dunes with a distinct slip face and two wings pointing downwind.	Fig.S2 C	3-5; 7-9
		Compound transverse megadune	Megadune with a distinct slip face and an asymmetric cross-sectional profile. The long longitudinal crest is per-pendicular to the unimodal wind direction.	Fig.S2 D	3-5; 10
		Complex transverse megadune	Megadune with a distinct slip face, formed by the superimposition of other types of dune (e.g., longitudinal, star, etc.) on a transverse bedform.	Fig.S2 E	3-5; 10
		Parabolic megadune	Fixed or semi-fixed dune with a "U-shaped" or "V-shaped" planform and two long trailing ridges stabilized by vegetation on the windward side.	Fig.S2 F	3-5; 11
2	Longitudinal megadune	Compound longitudinal megadune	Longitudinal megadune with a straight and elongated ridge, and a symmetric cross-sectional profile.	Fig.S2 G	3-5; 9;12
		Complex longitudinal megadune	Longitudinal megadune with different dunes superimposed (e.g., star dunes or transverse dunes).	Fig.S2 H	3-5; 9;12
≥3	Star megadune	Network megadune	Megadune with a lattice pattern in plan. Deformation forms include fish scale, honeycomb, etc.	Fig.S2 I	5;10; 13
		Stellate megadune	Megadune with several shorter sand ridges and an angular cone shape. Sand ridges radiate outward from the top of the dune.	Fig.S2 J	3-5; 13;14
		Pyramid megadune	Megadune with three triangular slopes and a broad, gently sloping base, resembling a pyramid.	Fig.S2 K	3-5; 13;14

Table S2. Dunefields\deserts with megadunes. The extent of main dunefields/deserts is obtained by tracing the dunefield area based on Google Earth images and DEM data within arid and semi-arid regions. The approximate distribution area and the megadune quantity of different types in each dunefield are listed.

Region		Desert		Central location		Megadune within the desert				
						Megadune field area (km^2)	Megadune quantity			
No.	Name	No.	Name	Lon(°E)	Lat(°N)		D_d	D_t	D_l	D_s
Ⅰ	North America	1	Mojave Desert	-115.99	35.84	45.77	-	-	2	4
		2	Great Basin	-115.68	39.85	5.15	-	-	-	1
		3	Sonoran Desert	-113.25	30.19	869.39	-	1	-	90
Ⅱ	South America	4	Acatama & Sechura Deserts	-73.92	-16.58	1212.25	-	17	30	95
		5	Monte Desert	-66.84	-35.35	765.78	-	64	-	-
Ⅲ	North Africa	6	Erg Trarza	-14.22	18.20	427.37	-	8	-	-
		7	Erg Aoukar	-9.53	17.39	3951.71	-	47	-	-
		8	El Djouf	-6.75	20.01	18018.68	-	3	61	-
		9	Erg Chebbi	-3.99	31.14	60.20	-	-	-	13
		10	Ergs Iguidi & Chech	-3.36	26.55	147497.07	-	-	696	4445
		11	Grand Erg Occidental	0.48	30.53	24595.03	164	783	-	141
		12	Erg Tamanghasset	5.25	24.61	8419.74	-	-	159	705
		13	Grand Erg Oriental	6.94	31.21	105662.44	862	-	487	10302
		14	Erg Issaouane	7.62	27.45	26191.38	-	248	317	618
		15	Idehan Ubari	11.83	27.15	49568.12	-	1469	234	3355
		16	Erg of Bilma	13.18	17.32	62373.32	-	285	181	427
		17	Idehan Murzuq	13.21	24.72	31397.22	225	-	516	1831
		19	Erg Al Jufrah	18.47	28.38	783.69	-	-	-	56
		20	Rebiana Sand Sea	19.94	26.32	5951.82	-	-	29	50
		21	Great Sand Sea	24.24	26.06	98352.70	-	496	290	23
		22	Qattara Depression Desert	28.67	29.46	7587.71	-	7	36	-
		23	Dakhla Farafra	28.67	26.61	2449.86	-	-	18	-
Ⅳ	South Africa	18	Namib Desert	14.33	-22.27	17745.48	-	-	394	810
Ⅴ	West Asia	24	Sinai & Negev Deserts	33.35	30.73	807.59	-	11	-	-
		25	An Nafud & Ad Dahna Deserts	43.56	25.93	34657.07	355	1947	117	30
		26	Sabatayn Sand Sea	46.54	15.46	1401.43	-	-	11	-
		27	Rub'al Khali Desert	50.8	20.95	172655.41	253	5799	1201	5801
		28	Kavir Desert	56.01	33.44	2057.04	-	87	-	9
		29	Wahiba Sands	58.82	21.64	2696.62	-	-	45	-
		30	Lut Desert	58.86	30.28	6497.40	-	141	100	286
		31	Registan Desert	64.06	30.35	9495.05	-	283	-	-
		32	Kharan Desert	64.12	28.23	1176.32	-	28	7	-
Ⅵ	Central Asia	33	Moiynkum Desert	71.00	44.07	1388.84	-	-	15	-
		35	Taukum Desert	75.44	44.74	538.57	-	3	14	-
Ⅶ	South Asia	34	Thar Desert	72.20	27.09	26854.94	-	148	-	-
Ⅷ	East Asia	36	Taklamakan Desert	83.59	39.09	58586.42	15	2059	-	28
		37	Xiaxiahete kumu Desert	85.88	47.94	584.53	-	-	-	97

		38	Gurbantunggut Desert	87.95	45.19	8216.06	-	89	-	9
		39	Shanshan Desert	90.24	42.66	1796.00	-	-	52	157
		40	Kumkol Desert	90.61	37.02	1274.59	-	34	-	162
		41	Kumtagh Desert	92.33	39.84	8855.44	-	18	146	277
		42	Qaidam Desert	94.30	37.59	637.23	-	84	-	-
		43	Mingshashan Desert	94.64	39.98	322.39	-	-	-	159
		44	Altan, Bor Khyariin & Mongol Els	94.72	48.68	2748.39	-	35	33	52
		45	Badain Jaran Desert	102.27	40.41	28557.93	-	316 8	26	1095
		46	Tengger Desert	104.24	38.60	4960.53	-	86	0	13
		47	Ulan Buh Desert	106.41	39.97	601.18	-	15	14	16

Table S3. Megadune fields with depressions in their bottom terrains. An investigation of the bottom terrain of global megadune fields showed that, the three megadune fields in the Idehan Ubari, Rub 'al Khali, and Badian Jaran deserts contain significant depressions. Columns 2 - 4 show the location, approximate depth, and approximate area of the depression in each dune field, respectively. Columns 6 and 7 respectively show the number of megadunes distributed in the entire dune field (Q1) and the number of megadunes distributed only in depressions (Q2) within a certain height range. The last column shows the proportion of the number of megadunes distributed in the depressions to the number of megadunes distributed in the entire dune field within a certain height range.

Megadune field	Depression parameters			Megadune Quantity			
	Location	Depth (m)	Area (km^2)	Height range (m)	Dune field region (Q1)	Depression region (Q2)	Q2/ Q1
Idehan Ubari Desert	9°20′E,27°35′N	75	3922	≥100	5058	999	0.20
	10°20′E,27°30′N			≥200	42	10	0.24
		48	5501	≥300	-	-	-
				≥400	-	-	-
Rub' Al Khali Desert	54°35′E,21°30′N	78	87343	≥100	13054	7993	0.61
				≥200	201	194	0.97
				≥300	-	-	-
				≥400	-	-	-
Badian Jaran Desert	101°14′E,40°5′N	39	1504	≥100	4289	323	0.08
				≥200	1332	282	0.21
	102°5′E,39°56′N	24	1242	≥300	373	162	0.43
				≥400	25	11	0.44

Dataset S1 (separate Excel file). Database of global megadunes. The dataset is an Excel file with 9 sheets. Sheet 1 contains basic information (including the serial number, longitude, latitude, and area) of each megadune field. Sheets 2 - 9 contain megadune datasets for North America, South America, North Africa, South Africa, West Asia, Central Asia, South Asia, and East Asia.

SI References

1. J. Laity, Deserts and Desert Environments (Wiley-Blackwell, 2008).
2. A. Gunn et al., What sets aeolian dune height? *Nat. Commun.* **13**, 1-8 (2022).
3. E. D. McKee, *A Study of Global Sand Seas* (US Geological Survey, 1979).
4. D. S. Thomas, *Arid Zone Geomorphology: Process, Form and Change in Drylands*, 3rd ed (John Wiley & Sons, 2011).
5. S. C. Du Pont et al., Complementary classifications of aeolian dunes based on morphology, dynamics, and fluid mechanics. *Earth Sci. Rev.* **225**, 104772 (2024).
6. A. S. Goudie, A. M. Goudie, H. A. Viles, Dome dunes: Distribution and morphology. *Aeolian Res.* **51**, 100713 (2021).
7. A. S. Goudie, Global barchans: A distributional analysis. *Aeolian Res.* **44**, 100591 (2020).
8. K. Pye, H. Tsoar, Aeolian Sand and Sand Dunes (Springer-Verlag Berlin Heidelberg, 2009).
9. N. Lancaster, Geomorphology of Desert Dunes，2 ed (Cambridge University Press,2023).
10. R. Cooke, A. Warren, A. Goudie, *Desert Geomorphology* (UCL Press,1993).
11. N. Yan, A. C. W. Baas, Parabolic dunes and their transformations under environmental and climatic changes: Towards a conceptual framework for understanding and prediction. *Global Planet. Change* **124**, 123-148 (2015).
12. C. Bristow, S. Bailey, N. Lancaster, The sedimentary structure of linear sand dunes. *Nature* **406**, 56–59 (2000).
13. I. Livingstone, A. Warren, *Aeolian Geomorphology: A New Introduction* (John Wiley & Sons, 2011).
14. A.S. Goudie, A. M. Goudie, H. A. Viles, The distribution and nature of star dunes: A global analysis. *Aeolian Res.* **50**,100685 (2021).
